# Analysis of severe hand trauma injury frequency during “Munich’s Oktoberfest” within the last 9 years in comparison to years with absence due to the COVID-19 pandemic

**DOI:** 10.1007/s00402-022-04745-2

**Published:** 2023-01-05

**Authors:** Elisabeth M. Haas-Lützenberger, Constanze Kuhlmann, Riccardo E. Giunta, Karl-Georg Kanz, Dieter Steinbrunner, Wendelin Rittberg, Viktoria Bogner-Flatz, Dominik Hinzmann

**Affiliations:** 1 Division of Hand, Plastic and Aesthetic Surgery, University Hospital, LMU Munich, Ziemssenstr. 5, 80336 Munich, Germany; 2 Department of Trauma Surgery, Klinikum Rechts der Isar, Technische Universität München, Ismaninger Str. 22, 81675 Munich, Germany; 3 EMS Authority of Munich, Ruppertstraße 19, 80337 Munich, Germany; 4 Department of Anesthesiology, Intensive Care and Pain Medicine, Unfallkrankenhaus Berlin, Warener Str. 7, 12683 Berlin, Germany; 5 Department of Orthopedics and Trauma Surgery, Musculoskeletal University Center Munich (MUM), University Hospital, LMU Munich, Ziemssenstr. 5, 80336 Munich, Germany; 6 Department of Anesthesiology and Intensive Care, Klinikum Rechts der Isar, Technische Universität München, Ismaninger Str. 22, 81675 Munich, Germany

**Keywords:** Hand trauma, EMS, Oktoberfest, IVENA, COVID-19, Hand surgery

## Abstract

**Introduction:**

Within its 2 week period, the Oktoberfest attracts around 6 million visitors yearly to Munich, Germany. Due to alcohol intake, congested halls, and disorderly activities, numerous accidents occur. Although many hand injuries are observed, the impact of Oktoberfest on severe hand trauma injury frequency is under investigated.

**Materials and methods:**

Data from the regional emergency medical service (EMS) was analyzed over a 9 year period regarding the frequency of severe hand injuries during the world’s largest fair and compared to the corresponding period in the years 2020 and 2021 where the event was cancelled due to the world-wide COVID-19 pandemic. Additionally, we compared the patient numbers during the same period in one Emergency Department of a level-1-trauma and hand trauma center located close to the venue.

**Results:**

An exploratory description is made from data collected over a 9 year period (2013–2021) with focus on hand injuries before, during and after the “Oktoberfest”. A total of 4017 hand injuries were allocated to hospitals by EMS. There was an increase in severe hand injuries by 66% during the 2-weeks-Oktoberfest-period compared to years where the Oktoberfest did not take place. Pre-pandemic statistics show an increasing severe hand trauma frequency of 57.5% in September, compared to EMS-referrals during the remanding year.

**Conclusion:**

The risk of injuring relevant structures of the hand during Oktoberfest is extremely high as compared to other parts of the body due to beer stein and fall-associated injuries. These injuries can lead to lifelong impairments. Our data are the first that quantifies and pinpoints the risk of severe injury to the hand during Oktoberfest and therefore, it is of great interest for visitors, hand surgeons, paramedics and emergency department healthcare workers.

**Supplementary Information:**

The online version contains supplementary material available at 10.1007/s00402-022-04745-2.

## Introduction

Hand injuries affect all demographic sectors of current population and have considerable socioeconomic impact in severe cases. Severe hand injury has consequences not only in regard to profession, but in almost all areas of life, including simple household tasks and leisure activities. Therefore, underestimating severity of hand injury can lead to an irreversible disablement. It is crucial to make a correct injury assessment at first patient contact and moreover to sharpen attention for these injury patterns. Often, clinical training in hand examination is very poor, so that in emergency departments where there is no hand surgeon on site, it is common for injuries to be underestimated or misdiagnosed. Although it is well-known that hand injuries are one of the most common reasons for presentation in a surgical Emergency Department (ED). Unfortunately, there is only few data in literature. Therefore, obtaining data of health care research is also politically of great interest. Referral figures via emergency medical service (EMS) do not reflect the total numbers, as the majority are self-referrals [1].

ED visits have certain peaks throughout the calendar year. Well known is the increase in visits, for example, during New Year’s Eve or boxing holidays [2]. A unique period in Munich is during the 16–18 days in September through the beginning of October. At this time of the year, the world-famous Oktoberfest takes place. The world’s largest fair attracts around 6 million visitors each year. The event has a huge economic impact, increasing hotel stays, restaurant sales, brewery receipts and providing 12,000 jobs. With a composition of at least 14% international visitors, our analysis of hand injury cases during that period is of great interest beyond Bavarian borders.

On a national and regional aspect, it is important for optimizing infrastructure and on an international aspect our data represent the risk of hand injuries for everyone while visiting a festival.

There is an overall increase of all ED referrals due to heavy drinking during Oktoberfest, thus we aim to analyze in which way the resulting amount and characteristics of hand injuries were affected compared to the rest of the year [3]. During the last 2 years (2020–2021) Oktoberfest had to be cancelled due to social restrictions following the global spread of SARS-CoV-2. We intend to analyze changes in numbers of hand trauma injuries of corresponding time period in years before and within the pandemic, when Oktoberfest did not take place. For optimizing emergency medical care infrastructure, thinking of increasing specialist during certain times of the year, obtaining clinically and preclinically data is necessary, therefore analysis of the nature, content and urgency of increased patient numbers are needed.

With our presented data, we also want to highlight the high risk for visitors suffering from a beer stein injury resulting in potential lifelong disablement, due to injury of relevant functional structures of the hand.

## Materials and methods

### Emergency medical service data collection

Since 2013, the web-based IT system “IVENA” (Interdisciplinary Medical Care Capacity Management System) (IVENA eHealth, mainis IT-Service GmbH, Offenbach am Main, Hesse, Germany) is implemented to coordinate all hospital referrals via a Central Command Centre in Munich. The area covered by the Munich EMS (and thereby IVENA) includes around 1000 km^2^ and approximately 1.8 million inhabitants. The IVENA system collects basic information (patient age and sex, expected diagnosis (e.g. “hand trauma”) and medical specialty, triage category, medical transportation system and estimated time of arrival) and allocates the patient to a hospital, depending on proximity, availability and required resources (e.g. stroke unit, hand trauma center) [4]. When a patient is allocated, the hospital receives a web-based notification in real-time. There is an overall number of approximately 140,000 allocations per year in the defined region. In total, over one million allocations can be evaluated until now. Therefore, it is a unique data source of a city of millions of inhabitants.

For the analysis of the frequency of severe hand injuries, a retrospective analysis of data collected by IVENA ehealth from 1 February 2013 to 31 December 2021 was performed for all adult persons, that had been recorded as hand trauma by the EMS independent of insurance status. All cases that were referred to the hospital by paramedics were classified preclinically as “severe” hand trauma and required a medical transportation system. The term “severe hand injury” is considered to be so severe for the patient that he or she cannot get to the emergency room independently. It can be either a bony or deep soft tissue injury, most of them open and with severe bleeding. We then focused specifically on the time span during the 2 weeks of Oktoberfest before lockdown (years 2013–2019) and within the span of the pandemic (2020 and 2021) and compared additionally a 2 week period before and after the Oktoberfest regarding hand trauma cases.

### Single-center emergency department data collection

For an exemplarily description of the quantitative development of hand injuries during Oktoberfest, we analyzed data of hand trauma injuries of a level-1- trauma hospital and hand trauma center (City Center Hospital, University Hospital, LMU Munich, Bavaria, Germany) which is the closest hospital located next to the venue. Therefore, a retrospective analysis of patients’ medical records and the documentation software of the emergency department (since 2019: Epias, Epias GmbH, Idstein, Bavaria, Germany) was performed to identify all surgical patient admissions and all hand injuries during official Oktoberfest time according to each year. All surgical patients (including occupational injuries) from age 18 and all types of admissions (self-referral and EMS-referral) were included in this study. The daily hand trauma frequency was additionally calculated for a 2 weeks before- and after-period and were compared to the Oktoberfest period for the “Oktoberfest years” (2018–2019). Additionally we analyzed the most common types of injuries  that presented during Oktoberfest.

### Ethics statement and statistical analysis

The study was reviewed and approved by the Institutional Review Board of the medical faculty of the LMU Munich under the registration number 20–258 and was conducted in accordance with the Declaration of Helsinki. All data were imported to Microsoft Excel (Microsoft Corporation, Redmond, Washington, USA) for further analyses. Descriptive statistics were used for the calculation of the rates and means for the IVENA data. For statistical analyses of the hospital data either an unpaired *t* test (student’s test) or a Mann–Whitney *U* test were used after testing for Gaussian distribution. A *p* value of < 0.05 was regarded as statistically significant and results are presented as the mean ± standard deviation (SD). Graphs were designed with Microsoft Excel and GraphPad Prism 8 (GraphPad Software, La Jolla, California, USA).

## Results

### Development of hand surgery admissions per year

**Fig. 1:**
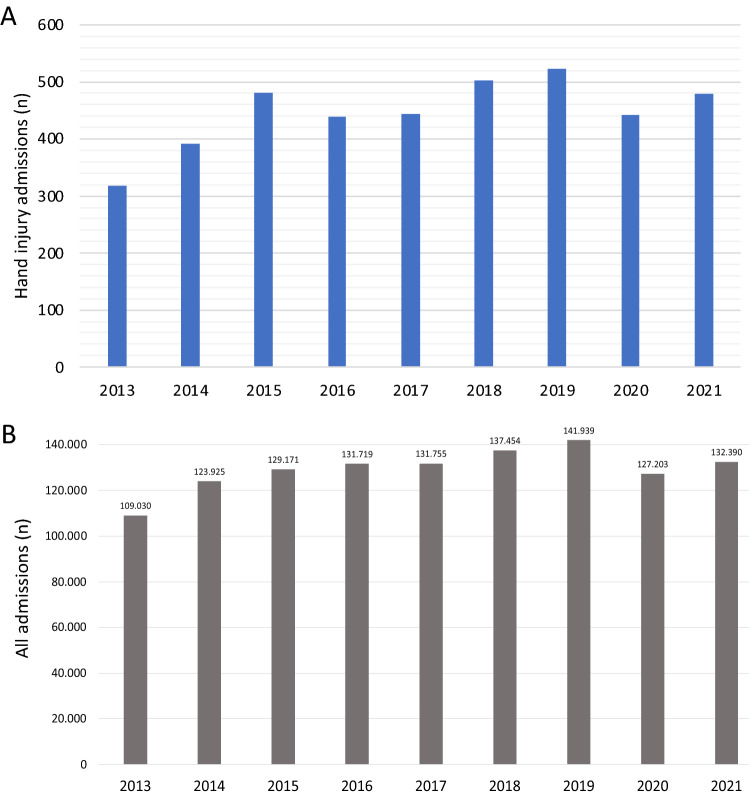
**A** shows the development of hand injury admissions by EMS. **B** shows the overall numbers of all Emergency Referrals (internal and surgical cases) in the greater Munich area from 2013 to 2021

A total of 4017 hand injuries were allocated to hospitals by EMS in a 9 years observation period from 2013 to 2021. Hand surgery ED admissions have continuously increased but showed no statistical linear function over the study time period from 2013 to 2019 (Fig. 1A). In 2013, IVENA rollout was only in February, so January numbers are incomplete. On average (mean), 446 severe hand surgery patients per year have been admitted to hospital by EMS. These have been diagnosed and categorized as “severe” hand injuries, as a classified minor injury would present as self-committal in ED. In 2020, the first year of the pandemic, there were 442 cases, a decrease of 15.5% from 523 compared to 2019. In 2021, numbers showed increase again to 479.

The total number of EMS admissions in Munich steadily increased within the last years before COVID-19 pandemic (Fig. 1B). Yet the mean in year 2020 and 2021 did not change significantly compared to mean referrals 2013 to 2019. Interestingly, in 2019 there was the highest peak recorded with a total of 141,939 referrals, and thus an increase of 3% compared to the previous year 2018.

### Demographic data and transportation systems

**Fig. 2:**
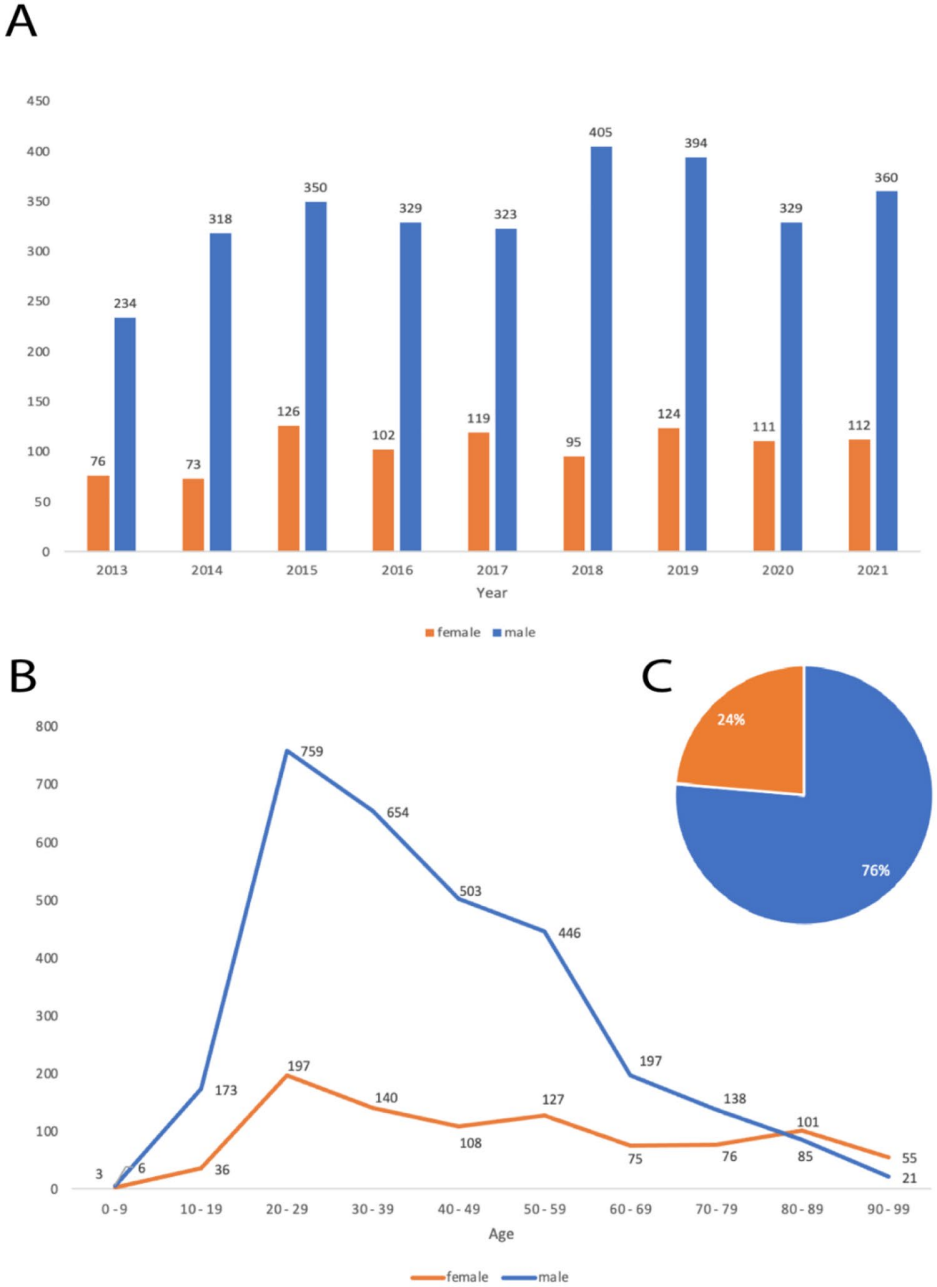
Demographic data of IVENA-registered hand injuries. “**A**” shows the gender distribution among the patients with hand injuries over the years 2013–2021. The age distribution among each gender is illustrated in “(**B**)”. On average, 76% of patients with registered hand injuries in Munich were male (**C**)

**Fig. 3:**
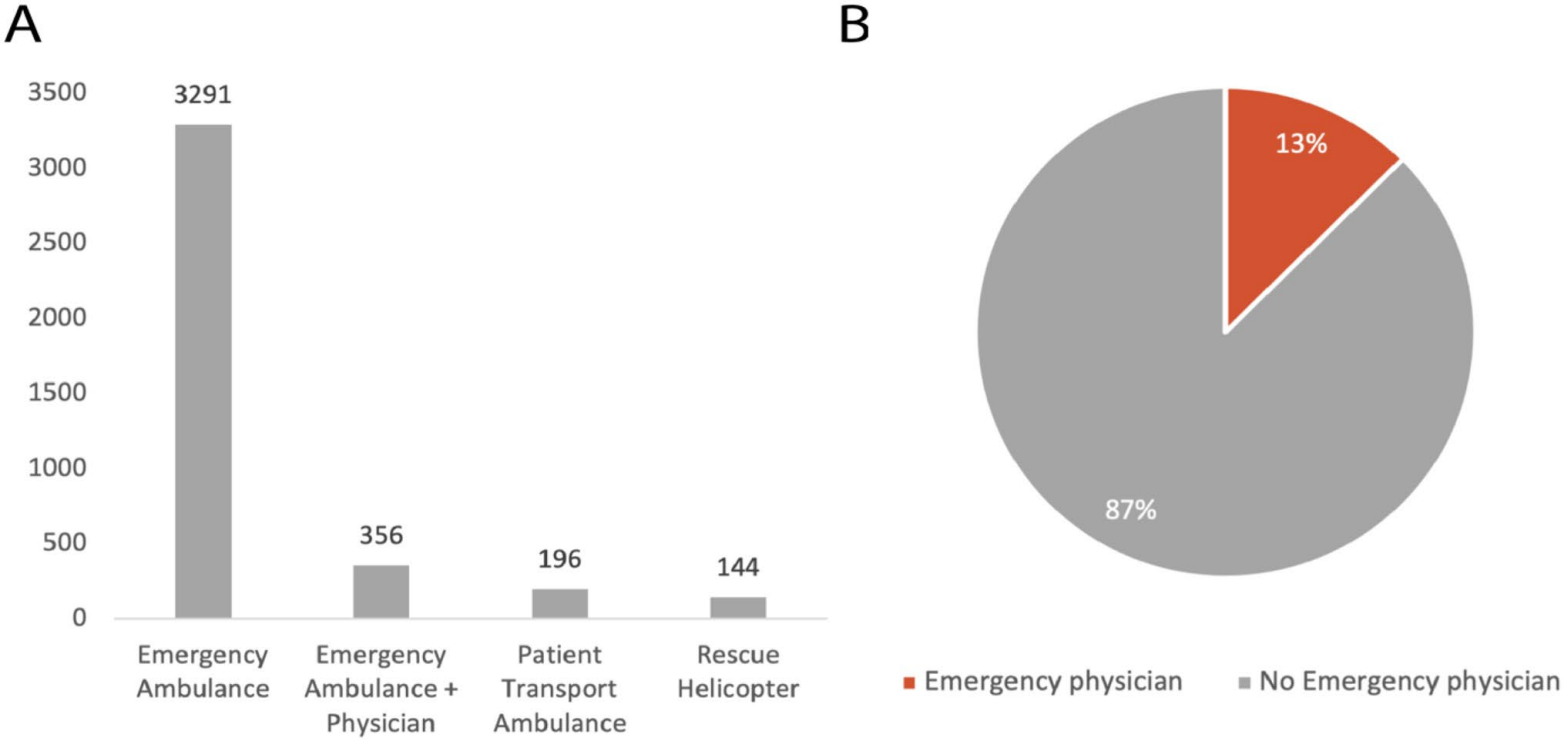
Transportation systems that were used for hand injury EMS-referral in Munich (**A**). 13% of EMS-referrals were accompanied by an emergency physician (**B**)

Analysis of the sex distribution of IVENA-registered hand injuries show that on average 76% of the patients were male. Further analysis of patients age shows an average age of 42 (± 17.7) years for male and 51 (± 23.6) years for female patients. Peak age for the occurrence of a hand injury was found to be in 20- to 29-years-old patients (Fig. 2). Furthermore, we analyzed the transport systems that were used for the EMS-referral of hand traumas from the scene to the hospital. Among all EMS-referred hand traumas, 96.4% of patients were transported with ground emergency medical services. The wide majority (82.5%) of hand traumas were assigned by ambulance with monitoring by paramedics. 3.6% of hand trauma patients were transferred to the hospital by air with a medical helicopter (Fig. 3A). 13% of EMS-referrals were accompanied by an emergency physician, while the remaining 87% of patients were transferred by paramedics (Fig. 3B).

### Development of hand surgery admissions during Oktoberfest

**Fig. 4:**
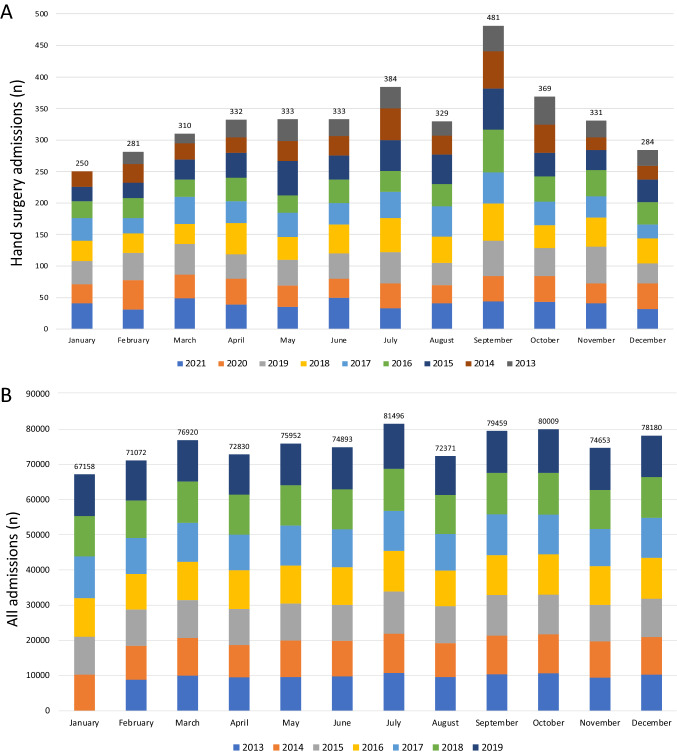
Numbers of hand surgery admissions presented by month (2013–2021) (**A**) and numbers of all Emergency Cases per months in the pre-pandemic period from 2013 (January is missing) to 2019 (**B**)

To identify the differences throughout the year, we describe the hand injury cases per month, to show the significant increase of total numbers during different seasons of the year. We mainly focused on the month “September” when the “fifth season”—how Bavarians nickname the 17 days of Oktoberfest—mainly take place. However, the number of hand surgical admissions decline in winter months. The month of September includes around 70% of the Oktoberfest days. If months January (excluded due to missing data of 2013) and September (due to Oktoberfest) were excluded for calculation of the average number of hand surgery referrals per months, there are 329 patients with hand injuries admitted to hospital by EMS in Munich. The highest number of hand surgery EMS admissions regularly occur in September with a total of 481. That means an increase of 46.2% in September compared to average referrals of the rest of the year (Fig. 4A). If we exclude data from the pandemic years, this trend becomes even more noticeable with an increase of 57.5% in severe hand trauma frequency which equals in 21 additional patients compared to the other months. Regarding the annual trend in the pre-pandemic period from 2013 to 2019 with focus on the overall number of all emergency referrals, there is a minor trend, but not a remarkable increase seen in months September and October (Fig. 4B).

**Fig. 5:**
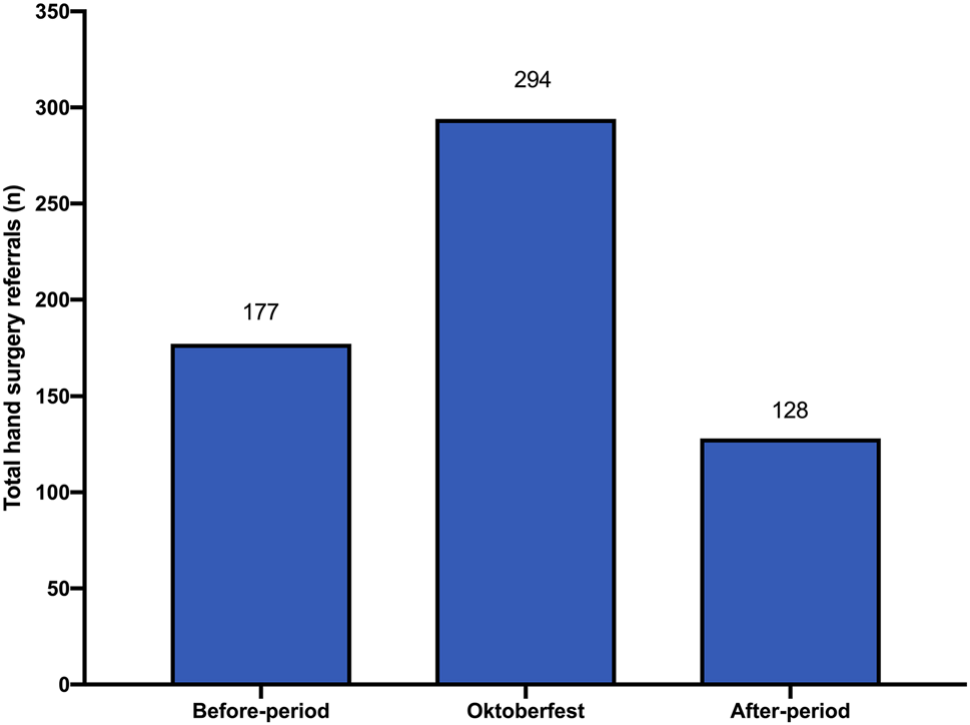
Comparison of hand surgery referrals of a 2-week-period before, during and after Oktoberfest (2013–2019). Total numbers of years 2013 to 2019 were depicted

Figure 5 shows the incidence of hand surgical ED admissions by EMS service of 17 days as this is the average duration of Oktoberfest and compared all hand surgery referrals during the Oktoberfest with the same time span before and after for the years 2013 to 2019. Compared to the period before the Oktoberfest, there has been an increase of 66% in the total number of hand trauma during Oktoberfest and a decrease of 27.7% in the period after.

### Surgical admissions in a single-center ED during Oktoberfest

**Fig. 6:**
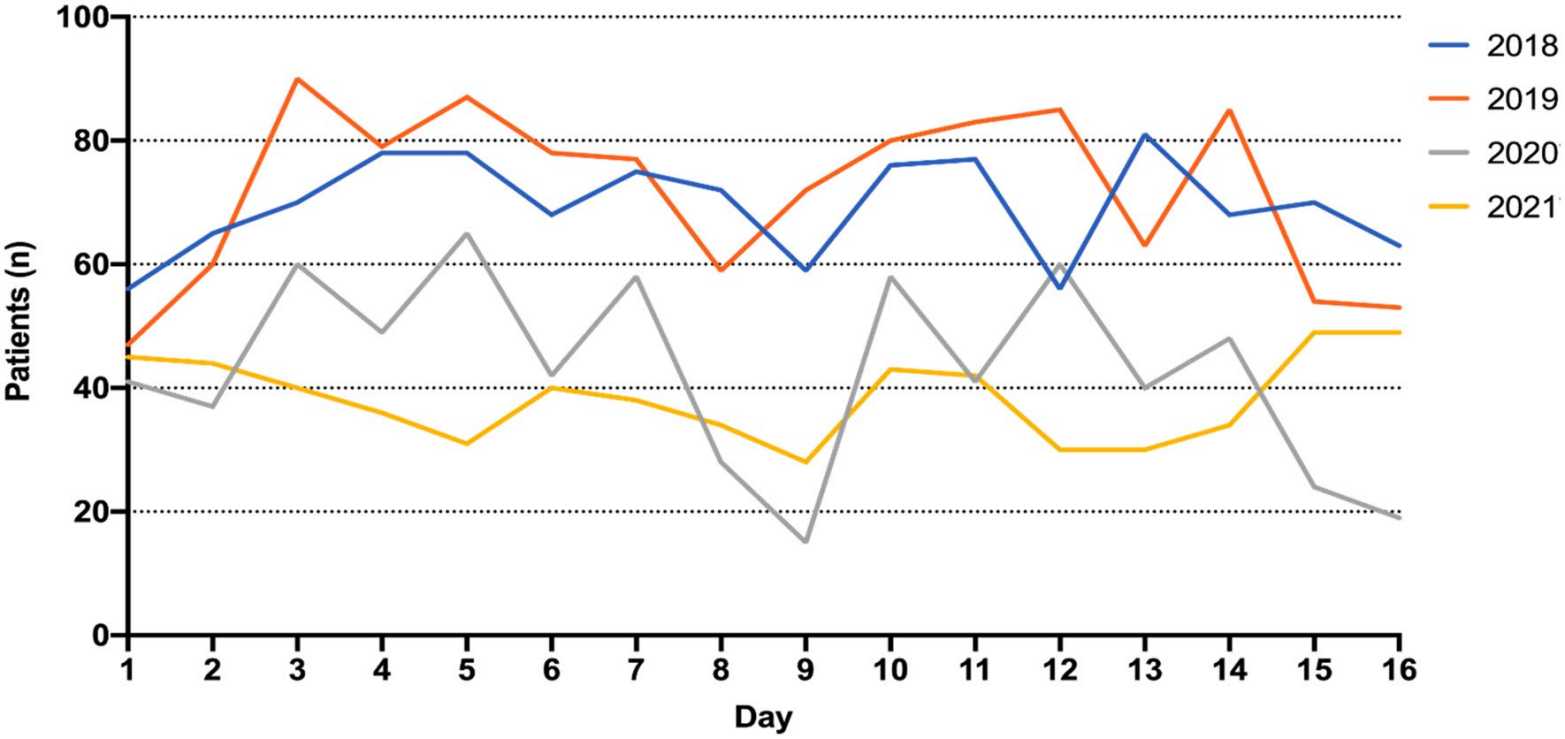
Numbers of daily surgical admissions (*n*) of one single center Emergency Department located in the City Center during 16 days of Oktoberfest (2018 + 2019) compared to non-Oktoberfest years (2020 + 2021) (self-referral and EMS-referral)

In addition to the data obtained from IVENA, we analyzed data obtained from a level-1- trauma hospital and hand trauma center (LMU Munich, City Centre hospital) which is located closest to the venue. Figure 6 illustrates the numbers of surgical ED admissions to the hospital in the years of the pandemic (mean 40.5 ± 11.94 patients) in comparison to the Oktoberfest years 2018 and 2019 (mean 70.75 ± 11.2 patients).

**Fig. 7:**
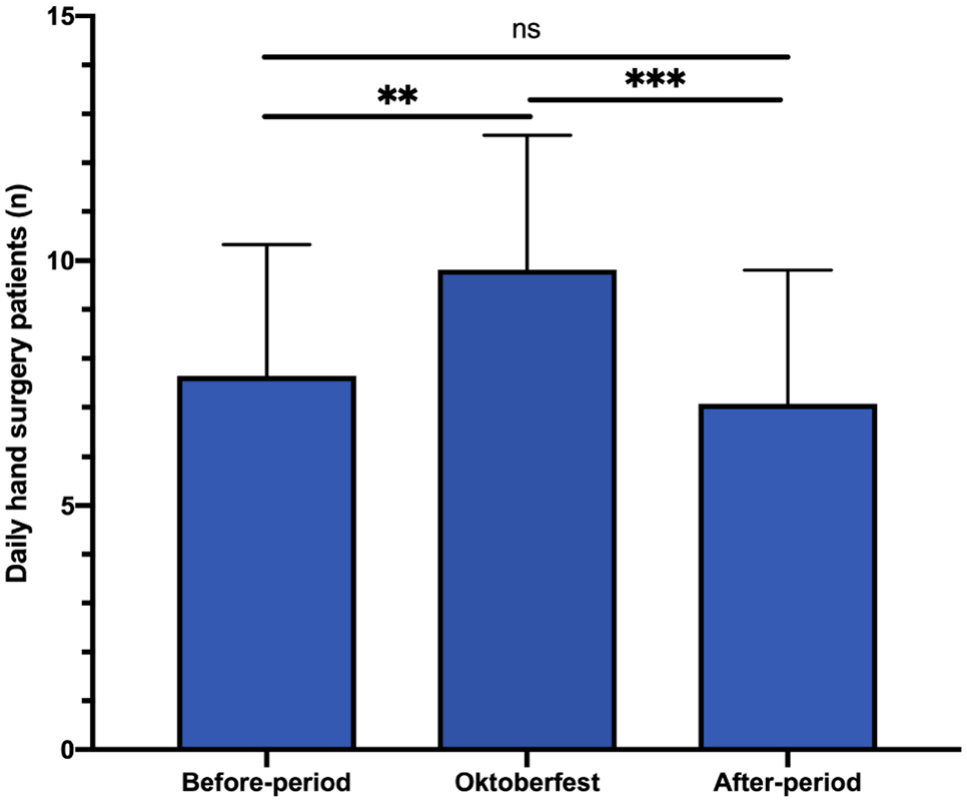
Number of daily hand surgery admissions in 2018 and 2019 to one single center Emergency Department (City Center LMU Munich) during Oktoberfest in comparison to a 2 weeks before and after period. During Oktoberfest the number of hand surgery patients was significantly higher. (ns = not significant; **p < 0.01; ***p < 0.001 mean ± SD)

We compared the number of daily hand surgical patients in our ED during a 2 week period before, during and after Oktoberfest for 2018 and 2019, similar to the IVENA data in Fig. 5. During Oktoberfest a significantly higher frequency of daily hand surgery admissions in comparison to the 2 weeks before (*p* = 0.0031) and after period (*p* = 0.0003) could be found (Fig. 7). In comparison to the before-period the number of daily patients increased by 28%.

**Fig. 8:**
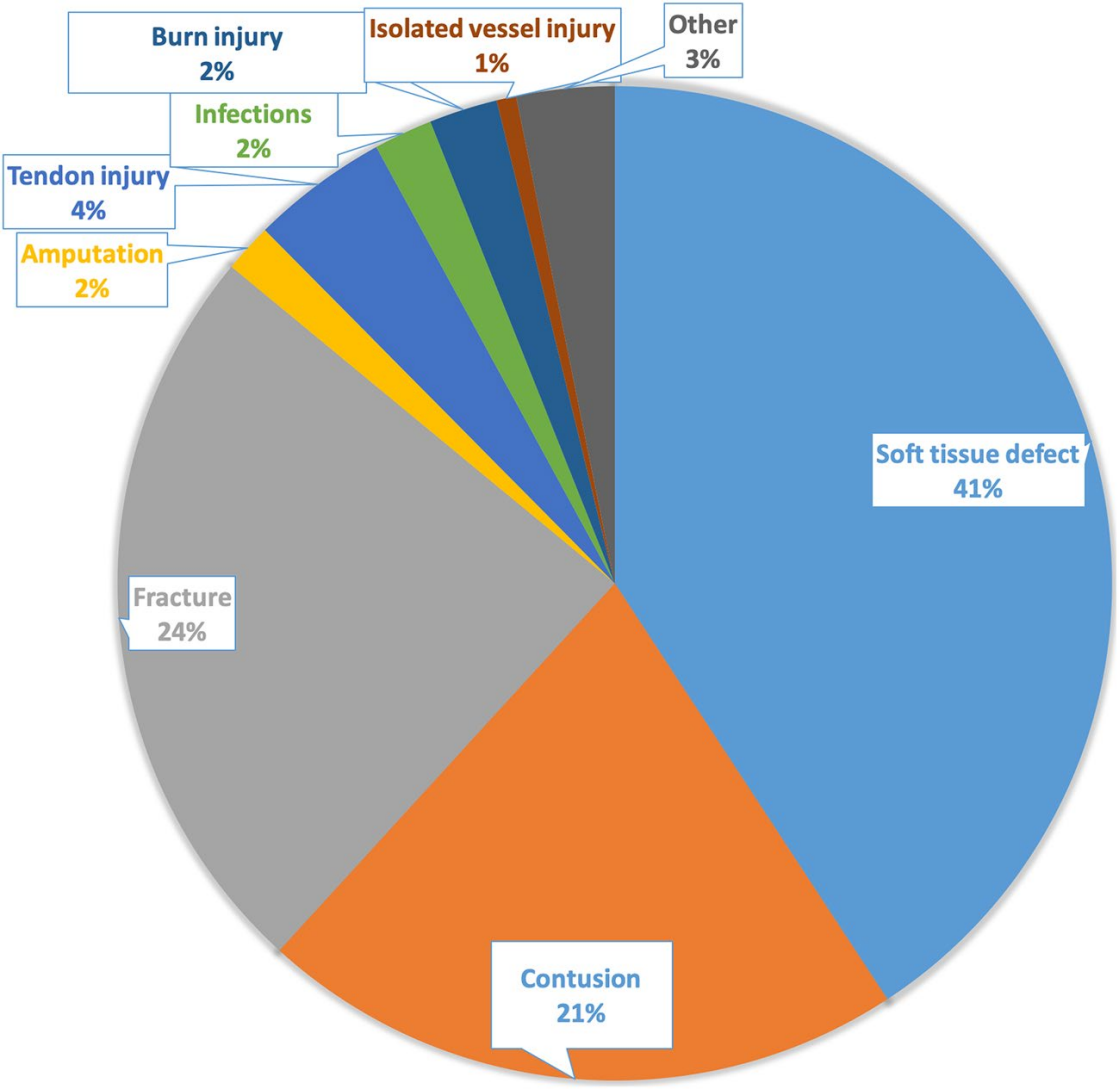
Most common injury types of hand injuries diagnosed in the ED within the Oktoberfest period in 2018 and 2019

Furthermore we analyzed the diagnoses coded for the types of hand injuries. Most common diagnoses were soft tissue injuries with a percentage of 41%, followed by fracture (24%) and contusion (21%) of the hand. Amputations, tendon injuries and isolated vessel injuries were occurred with a small percentage of 4, 2 and 1% (Fig. 8).

## Discussion

Hand injuries are known to be one of the most common surgical presentations in ED. However, the frequency of hand trauma in preclinical EMS is under investigated. The web-based IVENA system, that was implemented in 2013 to coordinate all hospital referrals in Munich provides a unique opportunity to analyze the prevalence and demographics of severe hand trauma throughout the year(s), with special attention to the Oktoberfest and COVID-19 pandemic.

Our analyses of a 9 year period (2013–2021) of data of IVENA system concerning numbers of hand injuries made up a total of 4017 out 1,164,586 total emergency referrals cases, providing a first percentage (0.35%) on frequency of hand trauma in preclinical EMS in Munich. Clearly, there is a huge gap between the number of preclinically registered hand injuries and the frequency in ED, where earlier studies from the United States and Germany found hand and wrist trauma the second most common diagnosis [1, 5]. The frequency of hand injuries in ED is described with large discrepancies ranging between 9 and 27% in literature and ranging between 13 and 15% in our surgical reference ED before the pandemic [6–8]. There is a lack of data, but these data are of great importance for organizing staff in EDs. As most hand injuries are self-referrals, gathering reliable total preclinic numbers of hand injuries of any severity is hardly impossible and leading to the conclusion that absolute number of all hand injuries remains unknown but certainly is ways higher in total than the cases presented here. In literature there are only few statistics on hand injuries based on national data. This is due to the lack of national registers. Not only regarding hand injuries, but it is also difficult to document recreational accidents. According to the most recent accident statistics from 2015, the Federal Institute for Occupational Safety and Health of German confirms that accidents that occur during leisure activities or in the household, account for a total of 72% (7.05 million) out of a total of 9.73 million accidents [9]. The problem is to obtain valid data as home and leisure accidents can only be estimated, for example, by the Robert Koch Institute (RKI) from survey data [10]. However, since IVENA registers all hand injuries, that require EMS-transportation, the presented data provide a realistic representation of severe hand trauma prevalence in the metropolis.

Considering the demographic data of IVENA-registered patients, males were significantly more likely to experience a severe hand or wrist trauma, accounting for 76% of EMS-referrals in Munich during the observation period. Overall, we found male adults aged between 20 and 39 years the most frequent demographic, revealing similar results as described in a retrospective national analysis of ED visits in the United States [11]. Previous studies demonstrate a variety of reasons for the discrepancy in hand injury rates between females and males, reporting a higher incidence of traumatic hand injuries in male athletes, rock climbers and amateur cooks [12–15]. Further analysis of the transportation systems, that were used for EMS-referrals revealed that most patients with severe hand trauma were transferred by ground emergency services (96.4%) without an emergency physician (87%). As hand injuries are rarely life threatening, only a small minority (3.6%) required air-bound hospitalization, which is usually reserved for ischemia-critical amputation injuries.

Oktoberfest marks a special highlight in the calendars of Munich’s first responders and ED employees. A recent IVENA-based study by Ghada et al. calculated an additional daily admission of 78 patients during Oktoberfest in the years 2014–2018 [16]. Within that number surgical admissions were slightly higher represented than internal cases and young male adults were at highest risk for an ED admission [16, 17]. Due to overcrowded halls and highly elevated alcohol intake (in total 7.3 million liters of beer in 2019) the predicted risk of injuring relevant structures of the hand is high due to exposed placement. Our data show an increase of 66% in hand trauma related ED admissions during Oktoberfest in comparison to the 2 week before-period. Furthermore, the month September (accounting for approximately 70% of Oktoberfest days) has the highest prevalence of severe hand trauma in the greater Munich area. Although the number of temporarily inhabitants of Munich increases, the number of hand trauma increased unproportionally. In comparison to the rest of the year, additional 21 cases of severe hand traumas occur in September. As stated above, those numbers only represent IVENA-registered hand trauma, and most hand injuries are self-referrals. Furthermore, during Oktoberfest most injuries are first examined in a sanitation station located at the venue, and emergency physicians decide who needs to be referred to EMS for further treatment in a hospital and who can be treated on site. That means that all minor (hand) injuries were treated directly without additional examinations in a hospital and IVENA registration. During the Oktoberfest 2019, a total of 6592 people were cared for in the sanitation station. It is not registered how much of them were hand traumas. Therefore, the actual number of hand injuries is even higher, but percentage can only be estimated as there is no data collection thereof.

To further evaluate the effect of the Oktoberfest on the workload and capacity of a single ED and hand trauma center, we analyzed the numbers of all surgical trauma and hand trauma admissions for a hospital that is located 1 km close to the Theresienwiese, where the event is held. For the analysis, we compared the 2 weeks before and after the event for the years 2018 and 2019. The analyses of the daily hand surgery patient frequency in the ED during Oktoberfest in comparison with a 2 weeks before and after period in non-pandemic years revealed a significant increase in hand injuries during the event. By elimination of a COVID-related bias, through the exclusion of pandemic years, the effect of the Oktoberfest on hand injury frequency as observed in preclinical IVENA data, becomes also apparent for the clinical hospital data. In summary, during Oktoberfest the frequency of hand trauma increased by 66% in EMS and by 28% in the exemplary level-1 hand trauma center closest to the venue.

The analysis of the injury pattern (Fig. 7) showed that about half of the patients require acute wound care, although an additional first aid center is open at the venue. This leads one to conclude that most of the superficial wounds are already treated and those showed up at the ED were deeper or require a specialist. With an increase between 28 and 66% of hand injuries during that period an increase in staff and space is necessary.

Within Munich three Hand Trauma Centers are located that provide care for the vast majority of complex and severe hand injuries. Therefore, the increasing frequency of severe hand trauma during the fifth season leads directly to increasing patient volume and predicted chance for emergency hand surgery in those centers. Interestingly, the overall number of hand injury related EMS- and self-referrals decreased during the 2 week period following Oktoberfest to a significantly lower number that was reported for the 2 week before period. Possible explanations for this trend could be a weather-related decrease of sports and recreational accidents in October compared to August and early September. Furthermore, Oktoberfest marks the end of summer in Munich, which is followed by a significant reduction in social and physical activity as the population returns to a normalized day to day pace.

### Limitations

This study has several limitations. First, data from January 2013 and February 2013 are incomplete, as these months were enrollment months and thus the system was not activated city-wide. Second, all admissions that were transported by the EMS ambulance are counted. In general, it is often difficult to correctly assess the severity of injury preclinically even for experienced paramedics. Sometimes it only becomes apparent during surgical exploration. We included only patients who were initially identified as hand injuries by the responsible paramedics. All other referrals were not included; therefore, it can be assumed that our results are rather below the real values. Within the evaluation of IVENA, no further basic data on the patients was collected, therefore no statement can be made on the actual severity of the individual emergencies. The evaluation does not provide any information about the further course of the patients. Data on the type of discharge or the inpatient course is not available. Third, as stated above, we drew a comparison between two time periods in years where Oktoberfest took place in 2020 and 2021 when it was cancelled due to the pandemic. However, one must not forget that during the pandemic phase not only the Oktoberfest did not take place, but the entire social life was shut down, so that the general risk of injuries in certain areas decreased. A simplified summery of regional restrictions during that time period can be found in the supplementary.

## Conclusion

In our data more than 4000 hand trauma injuries were analyzed. In the current literature, there is a lack of hand trauma data. This is the first long-term report of the necessity and use of EMS for hand trauma in a metropolis. Data of hand trauma from a single center is rarely published, but data for an entire metropolis is not available. Moreover, to our knowledge, no work in the literature exists that focus on the incidence of hand trauma during special circumstances like the 2 week time period of the worlds famous fair “Oktoberfest”. We provide data of a 9 year period that shows a clear increase in hand injuries during this period. Although there is knowledge of the increase in injuries in general during the Oktoberfest, there is a lack of valid data portraying this specifically for hand injuries. With the new data, we want to highlight and draw attention to the annual predictable increase in hand trauma injuries, and at the same time encourage hospitals to prepare their emergency departments sufficiently in advance for this small, but predictable wave. Additionally, we want to emphasize that during a festival that concerns 7 million people every year, there is a high risk of severe hand injury. This implies the significant impact of this event concerning the healthcare system. We want also draw attention to all visitors, especially that of the young male individuals, to be careful and attentive while drinking and dancing on benches and holding a heavy beer stein in their hand, as carelessness can lead to lifelong disablement. Another benefit of the published data is improvement of knowledge and awareness of paramedics regarding hand injuries. To implement this on site, we are organizing training courses by hand surgeons to ensure that paramedics recognize a severe hand trauma injury and knows when to refer to a hand trauma center to prevent severe and irreversible disabilities.

## Supplementary Information

Below is the link to the electronic supplementary material.

Supplementary file1 (DOCX 15 KB)
