# Supplementary material for: Analysis of severe hand trauma injury frequency during “Munich’s Oktoberfest” within the last 9 years in comparison to years with absence due to the COVID-19 pandemic
Source: Arch Orthop Trauma Surg. 2023 Jan 5;143(7):4527–37. doi: 10.1007/s00402-022-04745-2 (PMC9815061; doi:10.1007/s00402-022-04745-2)
Supplement: Supplementary file 1 — Supplementary file1 (DOCX 15 KB) [file 402_2022_4745_MOESM1_ESM.docx]

**Supplementary data**

|  | 2018 | 2019 | 2020 | 2021 |
| --- | --- | --- | --- | --- |
| Date | 22. Sep. – 7. Oct. | 21. Sep.– 6. Oct. | 19. Sep. – 4. Oct. | 18. Sep. – 3. Oct. |
| Mean number (± SD) of new reported COVID-19 cases in Munich per day | - | - | 93.5 (± 11.6) | 245.8 (± 27.8) |
| Oktoberfest | Open. | Open. | Cancelled. | Cancelled. |
| Restrictions | None. | None. | Social distancing, 1.5 m social distance in public, face masks in public areas, max. 10 Persons together in one room | 1.5 m social distance in public, face masks in closed rooms, public and private events: Max. 1000 persons, 3G; Larger events (max. 25000 persons) with regulatory approval, face masks and hygiene concepts |
| Gastronomy | Open. | Open. | Max. 10 Persons, 1.5 m distance between tables, hygiene concepts, contact tracking | 3G, hygiene concepts, contact tracking |
| Nightlife | Open. | Open. | Closed. | September: Closed. October: 3G, hygiene concepts and contact tracking |
| Source |  |  | BayMBl. 2020 Nr. 533 (09-17-2020)  BayMBl. 2020 Nr. 535 (09-22-2020)  BayMBl. 2020 Nr. 562 (10-01-2020) | BayMBl. 2021 Nr. 615 (09-01-2021)  BayMBl. 2021 Nr. 710 (10-01-2021) |

***Table 1.*** *Overview of the official Oktoberfest dates, local incidences and a simplified summary of the restrictions of the Bavarian government in public areas, gastronomy and night life during the COVID-19 pandemic in Munich. 3G = fully vaccinated, tested or recovered.*
